# Identification of the role of sugar-sweetened beverages in the progression of a murine metabolic dysfunction-associated steatotic liver disease model

**DOI:** 10.3389/fnut.2025.1710267

**Published:** 2025-12-03

**Authors:** Yong-Qiang Li, Chen Huang, Jiawei Chen, Siqi Yang, Jiemin Cheng, Huiting Chen, Yongjian Zhou

**Affiliations:** 1 Department of Gastroenterology, The First Affiliated Hospital, The First Clinical Medical School, Jinan University, Guangzhou, China; 2 Department of Gastroenterology and Hepatology, Guangzhou Key Laboratory of Digestive Diseases, Guangzhou Digestive Disease Center, Guangzhou First People's Hospital, School of Medicine, South China University of Technology, Guangzhou, China

**Keywords:** MASLD, fructose, sugar-sweetened beverages, bioinformatics analysis, dietary interventions

## Abstract

**Background:**

Rising metabolic dysfunction-associated steatotic liver disease (MASLD) prevalence parallels increased sugar-sweetened beverage (SSB) consumption. Clinical studies suggest differential metabolic effects of fructose, glucose, and sucrose, yet their distinct roles in MASLD pathogenesis remain uncharacterized in preclinical models. This study aimed to establish a murine model to dissect the specific contributions of fructose, glucose, and sucrose to MASLD progression.

**Methods:**

This study establishes a murine model to dissect SSB-specific contributions to MASLD progression. Eight-week-old male C57BL/6N mice were fed a high-fat high-cholesterol (HFHC) diet with/without fructose-, glucose-, or sucrose-sweetened beverages for 10 weeks. Hepatic transcriptomic profiles were analyzed via microarray, followed by functional enrichment. Protein-protein interaction (PPI) network and single-cell analysis identify pathway perturbations and hub genes.

**Results:**

Fructose-SB supplementation, unlike glucose or sucrose, exacerbated HFHC-induced MASLD phenotypes, including elevated body weight, hepatic steatosis, glucose intolerance, and hepatocellular injury. Transcriptomics identified 2,195 fructose-specific differentially expressed genes (DEGs: 1,978 upregulated, 224 downregulated). Upregulated DEGs were enriched in thyroid hormone signaling, lysosomal activity, and autophagy, while downregulated DEGs implicated oxidative phosphorylation suppression. PPI analysis revealed key hub genes (Akt1, Stat3, Ctnnb1, Ep300) and mitochondrial components (mt-Nd4, mt-Cytb, Uqcrq) as central regulators of fructose-driven pathology. Fructose-SB uniquely accelerates MASLD progression in HFHC-fed mice through transcriptional reprogramming of metabolic and mitochondrial pathways. In mice fed a high-fructose diet, expression of key hub genes was elevated, particularly in Kupffer and endothelial cells, which were also enriched in proportion. These findings highlight fructose-specific mechanisms in MASLD pathogenesis and identify potential therapeutic targets for SSB-associated metabolic disorders.

## 1 Introduction

Metabolic dysfunction-associated steatotic liver disease (MASLD) is a chronic metabolic stress-induced liver disease in genetically susceptible individuals due to nutrient overload and insulin resistance (1). The disease spectrum includes simple steatosis, metabolically related steatotic liver inflammation, and related fibrosis and cirrhosis (2). With the prevalence of obesity and type 2 diabetes mellitus (T2DM), the global incidence and prevalence of MASLD are increasing (3). In the recent decade, MASLD is emerging as the most frequent chronic liver disease worldwide, which affects 30%−40% people worldwide (4).

In addition to genetic factors, dietary habits are the main factor in the development of MASLD (5). The phenomenon of high-fat, high-cholesterol foods being sold together with sugar-sweetened beverage (SB) is widespread globally. Consumption of sugar-SB has still increased, especially in developing countries, which is closely related to the increasing global epidemic of obesity (6). High-sugar diets may increase the risk of MASLD through various mechanisms, including promoting insulin resistance, impairing beta cell function in the pancreas, and altering the gut microbiome. A systematic review and dose-response meta-analysis show that consumption of sugary drinks is associated with a dose-dependent increase in the risk of NAFLD (7). However, the effects of different types of sugars on MASLD progression are controversial. A clinical study reported that only consuming fructose-SB, but not glucose-SB decreased insulin sensitivity and increased visceral tissue adipose deposition in overweight humans (8). Furthermore, hepatic *de novo* lipogenesis increased in health men consuming fructose-SB and sucrose-SB but not in those consuming glucose-SB (9). However, another publication showed that there was no significant difference between high-fructose and high-glucose diets regarding liver triacylglycerol levels or biochemical parameters in overweight men (10).

The objective of this study was to construct a mouse model and investigated the hepatic outcomes of a high fructose-, sucrose- and glucose SB intake in the baseline of high fat high cholesterol diet induced MASLD mouse model. Thus, the aim was (i) to compare the roles of different types of sugar intake-in a liquid form, on the progression of MASLD. (ii) To identify the possible molecular mechanisms for fructose-specific effects.

## 2 Materials and methods

### 2.1 Animals and induction of MASLD

Twenty-five male C57BL/6N mice aged 8 weeks were obtained from Guangdong Provincial Medical Laboratory Animal Center (license editor no. SYXK2021-0002) and kept under specific pathogen-free conditions with a 12:12 h light-dark cycle at 20 °C temperature and 50% humidity in Daoke company. After one week of acclimatization, mice were randomly divided into five groups of 5 mice each. The first group was fed with sugar-free and low-fat control diet (CD, #TP26354B). The second group was fed with high fat, high cholesterol, sugar-free diet (HFHC, #TP26304B) and tap water. The third to fifth groups were fed with HFHC diet plus 10% fructose, glucose, or sucrose in drinking water, respectively. These diet formulations were purchased from Trophic Animal Feed High-tech Co., Ltd. (Nantong, Jiangsu, China). Diet consumption and body weight were examined every two weeks. Each cage of mice was fed with the same calorie diet and the same volume of water with or without sugar. After ten weeks of feeding, mice were anesthetized with 2,2,2-tribromoethanol (0.2 ml/10 g body weight, intraperitoneal injection), and euthanized by exsanguination via cardiac puncture under deep anesthesia. Serum and liver tissues were collected for histopathologic, biochemical, and molecular analyses. All animal experiments adhered to the ARRIVE guidelines and were reviewed and approved by the Animal Ethics Committee of South China University of Technology and IACUC of Daoke Medical & Pharmaceutical Company (Guangzhou, China). The approval (number IACUC-DK-2024-03-01-03) was granted on March 1, 2024.

### 2.2 Glucose tolerance test and insulin tolerance test

To assess insulin resistance and glucose homeostasis, glucose tolerance test (GTT) and insulin tolerance test (ITT) was performed before sacrifice. In a GTT, 2.5 g/kg (body weight) of glucose is administered by intraperitoneal (IP) injection after 16 h fasting. Serial venous blood samples are collected, and glucose level was measured at 0, 15, 30, 45, 60, 90, and 120 min by Accu-Chek performa (#06454011, Roche, USA). In an ITT, 0.5U/kg of insulin is injected after 4 h fasting, and the change in blood glucose is then examined, as with a GTT.

### 2.3 Liver histology

Briefly, the right lobes of mouse liver were fixed in 4% neutralized paraformaldehyde for 24 h, embedded in paraffin, and sectioned in 8 μm thickness. The slides were stained in hematoxylin and eosin (H&E), and the images were obtained with a NIKON Eclipse 80i inverted microscope (Nikon, Tokyo, Japan).

### 2.4 Oil red O staining

The right lobes of mouse liver tissues were embedded frozen and sectioned into 10-μm sections using a Leica cryostat. The slides were fixed in 4% paraformaldehyde for 2 min and 0.5% Oil-red O solution (Sigma-Aldrich, O1516) was applied on each slide for 10 min at room temperature. The slides were washed twice and then alum hematoxylin was used for nuclei staining. Finally, the slides were mounted in glycerine jelly.

### 2.5 Serological analysis

Whole blood of mouse was collected from the heart and serum was divided by centrifugation at 3,000 rmp, 4 °C for 10 min. Serum levels of alanine transaminase (ALT), aspartate transaminase (AST), and high-density lipoprotein (HDL) were determined using specific assay kits on the Mindrai BS-480 automatic biochemical analyzer.

### 2.6 RNA-sequencing analysis

The liver tissues were processed for RNA extraction and sequencing by Gene Denovo Biotechnology Co. Ltd. (Guangzhou, China). Briefly, total RNA was extracted using Trizol reagent kit (Invitrogen, Carlsbad, CA, USA) and quality control was assessed on an Agilent 2100 Bioanalyzer (Agilent Technologies, Palo Alto, CA, USA). The resulting cDNA library was sequenced using Illumina Novaseq6000. The reads were assigned to the mouse reference genome (Ensembl_release110) using STAR. Principal component analysis (PCA) was performed on the transcriptomic data to visualize global gene expression patterns and sample clustering.

### 2.7 Differential gene expression analysis

Triplicate replicates of mouse liver tissue were performed for each group. RNAs differential expression analysis was performed by DESeq2 software between two different groups. The genes/transcripts with the parameter of false discovery rate (FDR) below 0.05 and absolute fold change ≥ 2 was considered differentially expressed genes/transcripts (DEGs).

### 2.8 Differential gene expression analysis

Triplicate replicates of mouse liver tissue were performed for each group. RNAs differential expression analysis was performed by DESeq2 software between two different groups. The genes/transcripts with the parameter of false discovery rate (FDR) below 0.05 and absolute fold change ≥ 2 was considered differentially expressed genes/transcripts (DEGs).

### 2.9 Functional enrichment analysis

Gene Ontology (GO) enrichment analysis and KEGG pathway analysis were performed to identify significantly biological processes and enriched metabolic pathways, respectively. GO has three ontologies: molecular function, cellular component, and biological process. GO enrichment analysis provides all GO terms that significantly enriched in DEGs compared to the genome background and filter the DEGs that correspond to biological functions. The calculated p-value was gone through FDR Correction, taking FDR ≤ 0.05 as a threshold. Heat maps were plotted using https://www.omicshare.com/tools/, an online platform provided by Gene Denovo Biotechnology for data analysis and visualization.

### 2.10 PPI network construction and hub gene identification

Protein-Protein interaction network was identified using String v10, which determined genes as nodes and interaction as lines in a network. The network file was visualized using Cytoscape (v3.7.1) software to present a core and hub gene biological interact in Omicsmart (https://www.omicsmart.com).

### 2.11 Quantitative real-time PCR

Total RNA from liver tissues was isolated using Trizol reagent and reverse transcribed by the Prime Script RT Reagent kit (Takara, Otsu, Shiga, Japan). Quantitative real-time PCR (RT-qPCR) was performed using SYBR^®^ Premix ExTaqTM (Takara, Otsu, Shiga, Japan) on the ABI Prism 7000 (Applied Biosystems). GAPDH was used to normalize gene expression. Primer sequences are shown in Table 1.

**Table 1:** Primers used for detection of mouse gene expression.

**Primer**	**Sequence (5^′^-3^′^)**
mFasn-F	GGAGGTGGTGATAGCCGGTAT
mFasn-R	TGGGTAATCCATAGAGCCCAG
mAcly-F	CAGCCAAGGCAATTTCAGAGC
mAcly-R	CTCGACGTTTGATTAACTGGTCT
mScd1-F	TTCTTGCGATACACTCTGGTGC
mScd1-R	CGGGATTGAATGTTCTTGTCGT
mAkt1-F	ATGAACGACGTAGCCATTGTG
mAkt1-R	TTGTAGCCAATAAAGGTGCCAT
mStat3-F	CAATACCATTGACCTGCCGAT
mStat3-R	GAGCGACTCAAACTGCCCT
mCtnnb1-F	ATGGAGCCGGACAGAAAAGC
mCtnnb1-R	CTTGCCACTCAGGGAAGGA
mEp300-F	TTCAGCCAAGCGGCCTAAA
mEp300-R	CGCCACCATTGGTTAGTCCC
mmt-Nd4-F	ATCTGCTTACGCCAAACAG
mmt-Nd4-R	CTATGTGGCTAACTGAGGAG
mmt-Cytb-F	CCATTTATTATCGCGGCCC
mmt-Cytb-R	TTGATCCTGTTTCGTGGAG
mUqcrq-F	CCTACAGCTTGTCGCCCTTT
mUqcrq-R	GATCAGGTAGACCACTACAAACG

### 2.12 Western blot

Mouse liver tissues were collected and homogenized in WB Tissue/Cell Lysis Buffer supplemented with protease inhibitor (#AIWB-012, Affinibody, Wuhan, China). Following centrifugation at 14,000 rpm for 15 min at 4 °C, the supernatant was harvested. Protein concentrations were determined using the BCA assay, and equal amounts of protein extracts were separated by 5%−20% SDS-PAGE. The resolved proteins were transferred onto PVDF membranes, which were subsequently blocked with TBST containing 5% skim milk. Membranes were then incubated with primary antibodies (dilution 1:1000) overnight at 4 °C. The primary antibodies used included: p-mTOR (1:1000; #5536; CST, Danvers, MA, USA), mTOR (1:1000; #2972; CST, Danvers, MA, USA), p-AKT (1:1000; #4060; CST, Danvers, MA, USA), AKT (1:1000; #4691; CST, Danvers, MA, USA), and β-actin (#AC026, ABclonal, Woburn, MA, USA). Following incubation with secondary antibodies (1:5000; #7074; CST, Danvers, MA, USA) at room temperature for 1 h, protein bands were visualized using enhanced chemiluminescence (ECL) detection.

### 2.13 Single-cell analysis

Single cell sequencing data were processed using the Seurat R package (v5.0.1). Quality control involved discarding cells exhibiting >15% mitochondrial gene content or containing fewer than 200 or more than 2,500 detected genes. To mitigate batch effects, the Harmony R package (v1.2.3) was employed. Dimensionality reduction and clustering were performed with uniform manifold approximation and projection (UMAP). Cell clusters were subsequently annotated according to marker genes reported in previous studies (11). The *AddModuleScore* function was further utilized to evaluate expression scores of hub genes across distinct cellular populations.

### 2.14 Statistical analysis

GraphPad Prism 8.0 software (La Jolla, CA, USA) was used for all statistical analyses. Differences were analyzed using two-tailed *t* test and were considered statistically significant at *p* < 0.05.

## 3 Result

### 3.1 Fructose, but not glucose and sucrose-SBs aggravated MASLD progression

**Figure 1:**
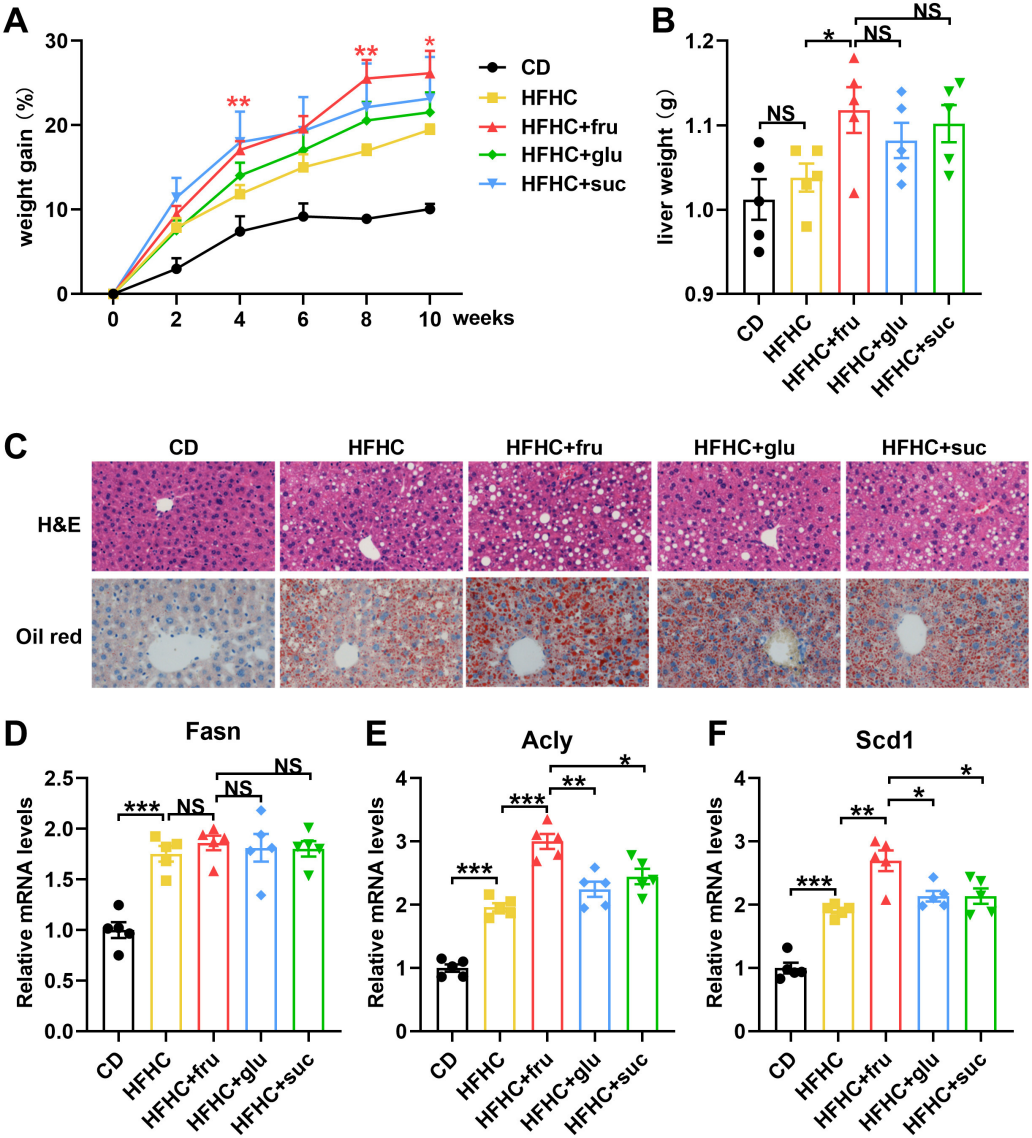
Fructose-sweetened beverage aggravates HFHC diet-induced hepatic steatosis. **(A)** Weight gain curve in every 2 weeks. *P* values were compared to the HFHC group. **(B)** Liver weight was measured. **(C)** Representative images of HE staining and Oil red staining. Images were taken at original magnification ( × 200). Scale bars, 100 μm. **(D–F)** The mRNA levels of Fasn, Acly and Scd1 were measured by RT-qPCR. *n* = 5. Data are mean ± SD. *p < 0.05; **p < 0.01; ***p < 0.001; NS, no significance.

To compare the effects of different types of sugar-SB consumption on MASLD progression, we constructed fatty liver model by high fat high cholesterol (HFHC) diet, and fed the mouse with fructose, glucose, or sucrose-SBs, respectively. After 10 weeks, the body weights of the HFHC and HFHC with sugar-SB groups were significantly higher than that of CD group. Compared to the HFHC, the groups consuming sugar-SB exhibited higher weight gain, especially fructose-SB consumption (Figure 1A). Similarly, the liver weight of HFHC with sugar-SB groups were also significantly higher than that of the CD group, whereas there is no significant difference among HFHC and HFHC with sugar consumption groups (Figure 1B). Figure 1C represented the histological characteristics and lipid deposition of liver in each test group. Consumption of sugar-SBs exacerbated lipid deposition. Mouse with beverage containing fructose exhibited the most serious lipid deposition of liver compared to the other two beverages. In addition, HFHC diet increased the expression of fatty acid synthase (Fasn), ATP-citrate lyase (Acly) and stearoyl CoA desaturase 1 (Scd1), which are critical enzyme for lipid metabolism (Figures 1D–F). Fructose induced higher levels of Acly and Scd1 than glucose and sucrose. The above data suggested that fructose-SB might exacerbate lipid deposition than glucose and sucrose-SBs.

**Figure 2:**
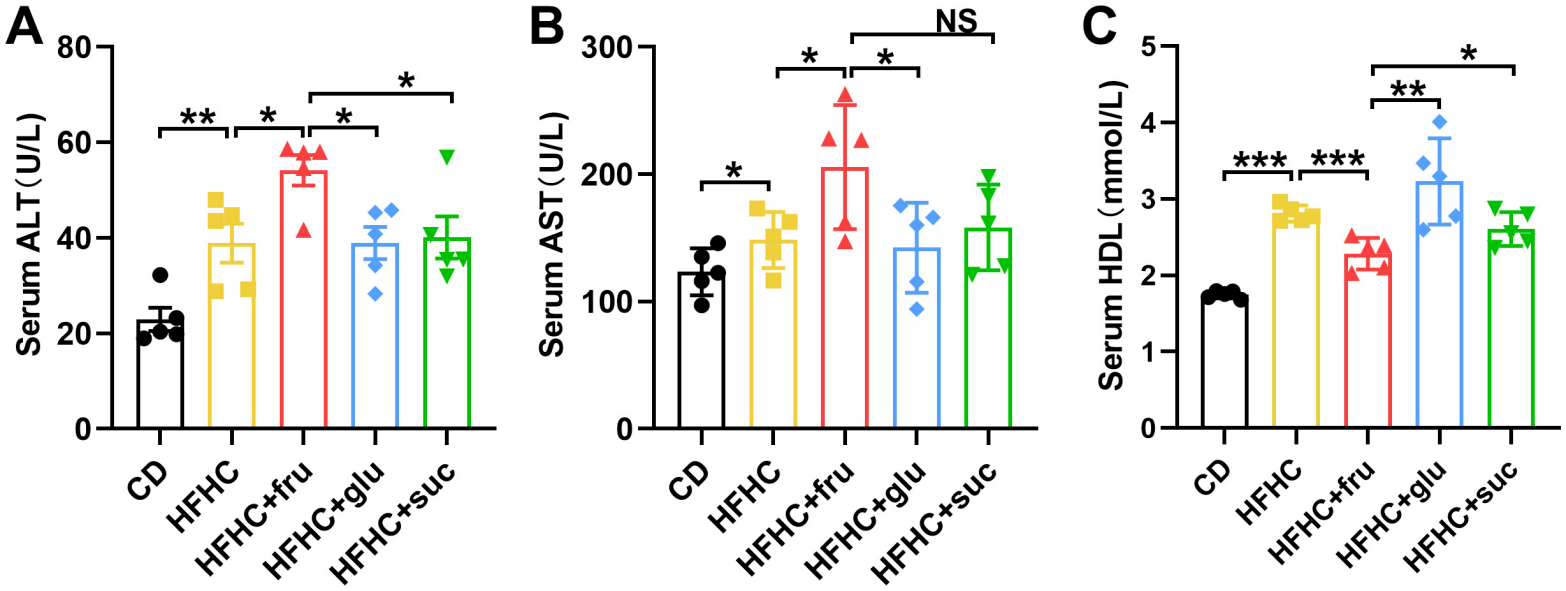
Effects of sugar-sweetened beverages on hepatic biochemical index. **(A–C)** Serum alanine aminotransferase (ALT), aspartate aminotransferase (AST) and high-density lipoproteins (HDL) were measured by colorimetric assay. Error bars are the mean ± SD, *n* = 5. *p < 0.05; **p < 0.01; ***p < 0.001; NS, no significance.

To investigate whether sugar intake would exacerbate liver damage in MASLD mice, we measured the changes in serum levels of alanine aminotransferase (ALT) and aspartate aminotransferase (AST) in each group of mice. As shown in Figures 2A, B, HFHC diet led to a marked increase in serum ALT and AST levels. Compared with the mice that consumed glucose or sucrose, the ones that consumed sucrose-SB exhibited higher ALT and AST levels, suggesting that fructose intake exacerbated liver damage. Interestingly, compared with the groups that fed with HFHC diet alone and those that consumed glucose or sucrose, the mice consuming fructose-SB exhibited lower high-density lipoproteins (HDL), which was reported to protect against hepatic lipid accumulation and atherosclerosis (Figure 2C). The above data indicated that consuming beverages sweetened with fructose, but not glucose and sucrose, increased liver damage.

**Figure 3:**
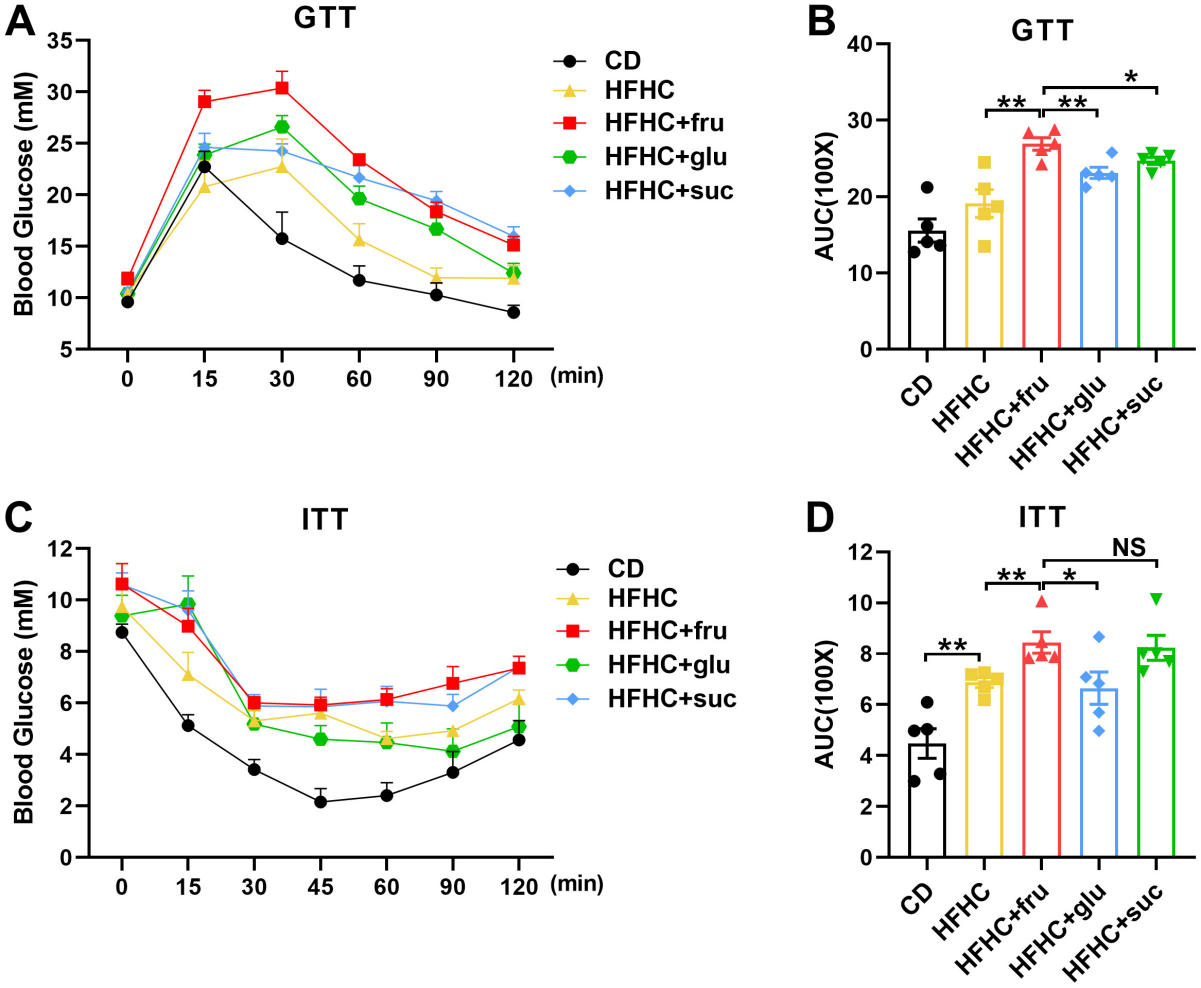
Effects of sugared beverages on glucose tolerance and insulin tolerance. Glucose tolerance test **(A, B)** and insulin tolerance tests **(C, D)** were performed in 10 weeks after HFHC diet and/or fructose, sucrose, glucose- sweetened beverage consumption. The corresponding area under the curve was calculated for each group. Error bars are the mean ± SD, *n* = 5. *p < 0.05; **p < 0.01; NS, no significance.

Insulin resistance is a determinant of progression of MASLD and is also one of the diagnostic indicators of MASLD. In order to investigate the different effects of fructose, glucose, and sucrose on glucose metabolism, we conducted glucose tolerance test (GTT) and insulin tolerance test (ITT) on mice. The results of GTT showed that there were no significant differences in fasting blood glucose levels among HFHC with or without sugar-SB groups (Figure 3A). However, the area under the curve (AUC) value of HFHC-fru was higher than HFHC alone, HFHC-glu and HFHC-suc, suggesting that consuming fructose-, but not glucose- and sucrose-SBs aggravated impaired glucose tolerance (IGT) (Figure 3B). In addition, compared the mice in HFHC and HFHC-glu groups, the ones in HFHC-fru group displayed enhanced insulin resistance (Figures 3C, D). Taken together, these data suggested that consuming beverages sweetened with fructose, but not glucose and sucrose, aggravated HFHC induced MASLD progression.

### 3.2 Differentially expressed genes (DEGs) screening for fructose-SB consumption

**Figure 4:**
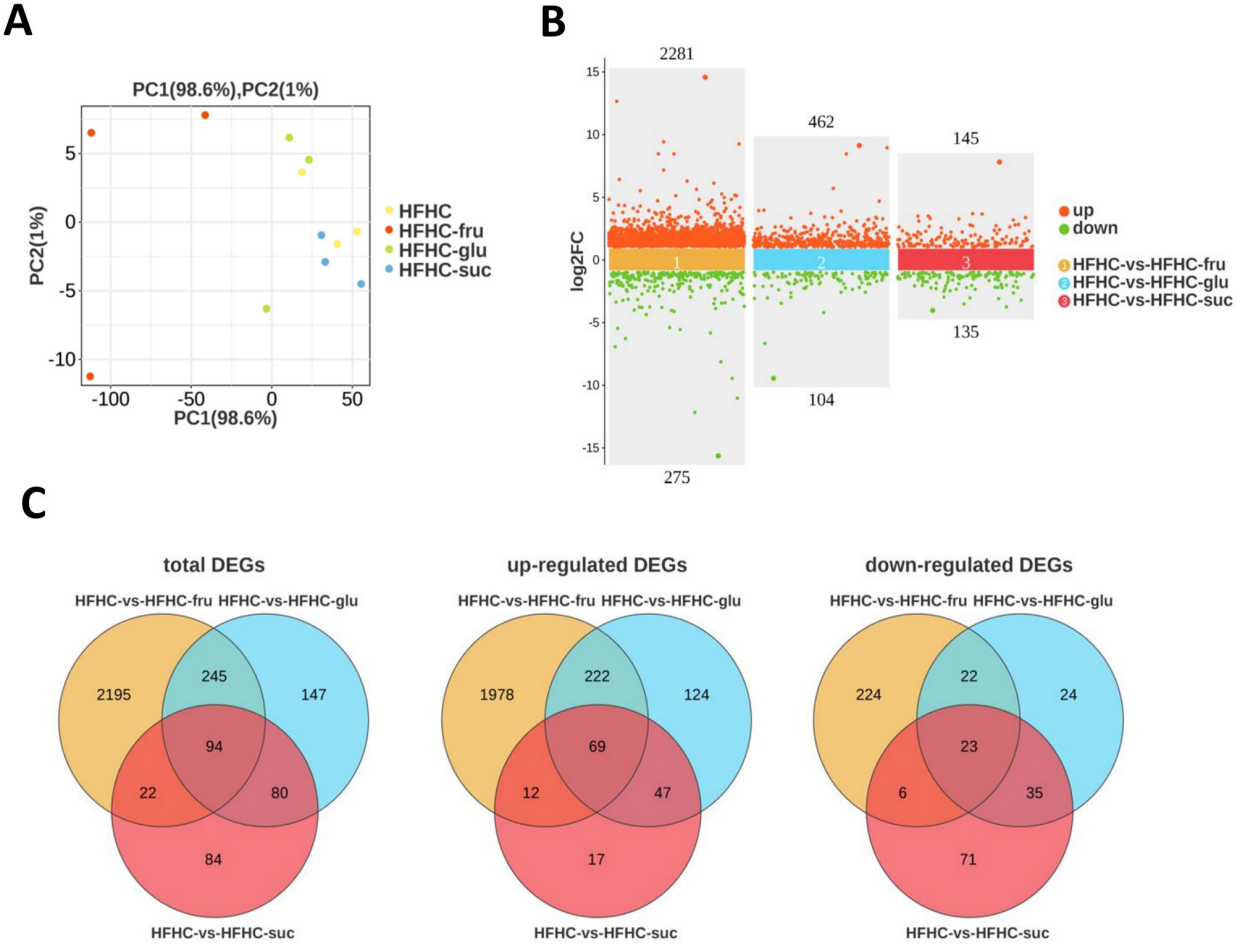
Changes of hepatic genes with or without sugar-sweated beverage consumption**. (A)** The relationship of each sample was analyzed by Principal component analysis (PCA). **(B)** Counts of genes up-regulated or down-regulated are shown in red or green dots, respectively (false discovery rate <0.05 and absolute fold change≥2). **(C)** Venn diagrams showed the numbers of differentially expressed genes among HFHC-vs-HFHC-fru, HFHC-vs-HFHC-glu and HFHC-vs-HFHC-suc datasets.

To investigate the molecular mechanism by which fructose differ from glucose and sucrose in MASLD progression, liver tissues of HFHC diet with or without sugar- SBs were subjected to RNA sequencing analysis. To analyze the relationships among each sample, we performed principal component analysis (PCA) based on the expression levels of the samples. As shown in Figure 4A, the liver samples from the HFHC-fru group are clearly separated from the HFHC alone, HFHC-glu and HFHC-suc groups, indicating that the transcriptome of the liver tissue in the HFHC-fru group is significantly different from the other three groups. Consistently, a total of 2,556 significant differentially expressed genes (DEGs), of which 2,281 genes were upregulated and 275 genes were downregulated in HFHC-fru compared with HFHC diet alone (Figure 4B). However, the numbers of up-regulated genes in HFHC vs. HFHC-glu and HFHC vs. HFHC-suc were just 462 and 145, respectively. Compared with HFHC diet alone, the numbers of downregulated genes in mice intaking glucose- and sucrose-SBs were 104 and 135, respectively. Furthermore, venn diagram analysis was used to find out distinct and overlapping genes among the three comparison groups. As shown in Figure 4C, a total of 94 DEGs were shared among the three treatments, whereas 2195, 147 and 84 DEGs were exclusively present in HFHC-fru, HFHC-glu and HFHC-suc groups, respectively. These results implied that these 2195 (1978 exclusively up-regulated and 224 exclusively down-regulated) DEGs might be responsible for aggravated HFHC induced MASLD phenotype under fructose-SB supplement.

### 3.3 Functional and pathway enrichment analysis of fructose-specific DEGs

**Figure 5:**
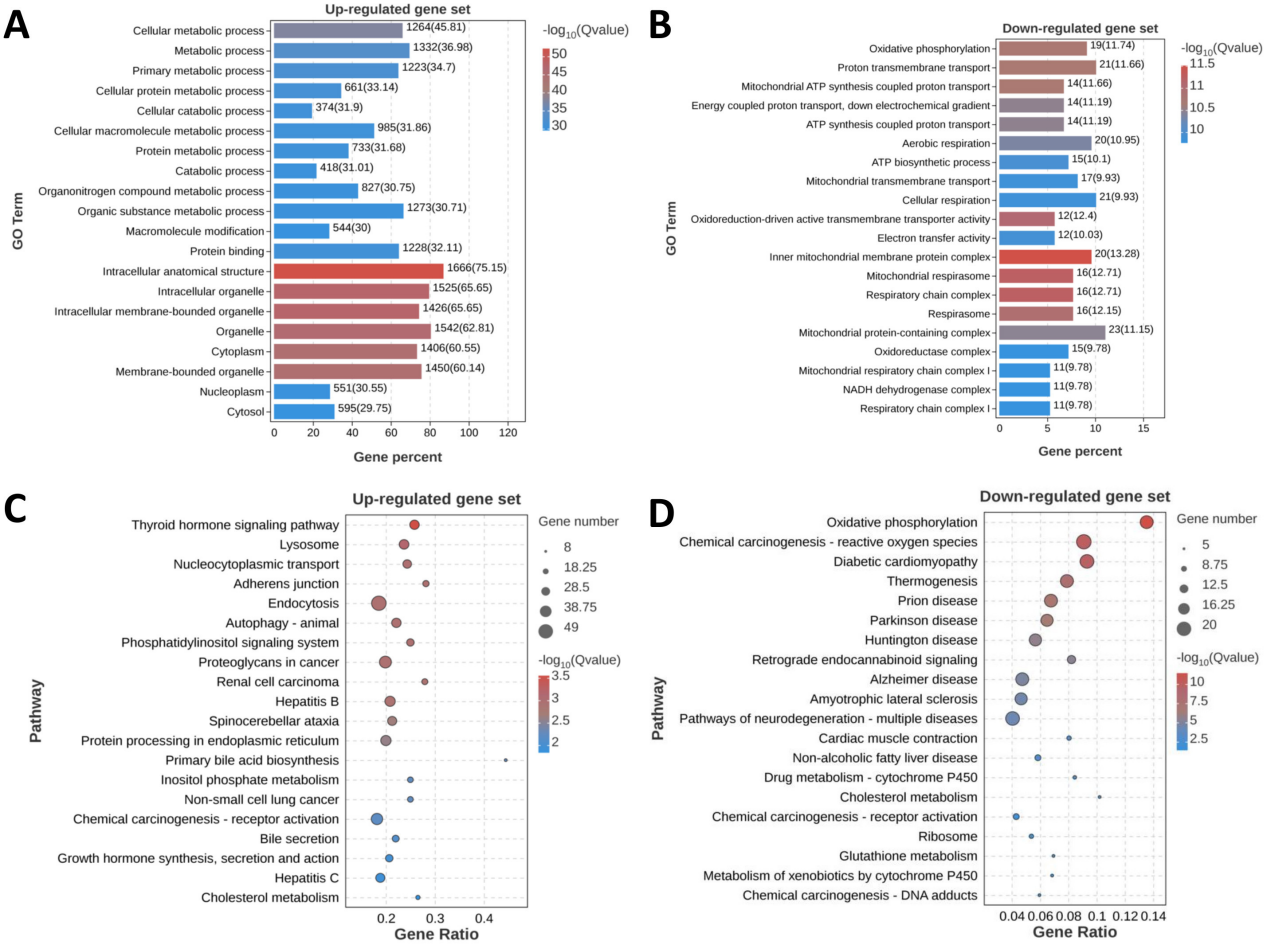
Functional and pathway enrichment analysis of fructose-specific DEGs. **(A, B)** Top 20 enriched gene ontology biological processes for fructose-sweetened beverage. Gene ontology enrichment analysis was performed on up-regulated gene set and down-regulated gene set, respectively. The significantly enriched gene ontology processes were ranked by their enrichment scores and the top 20 processes were shown. **(C, D)** KEGG pathways significantly enriched in up-regulated gene set and down-regulated gene set, respectively. The top 20 signaling pathways were shown.

To further explore the specific biological functions and signaling pathways interfered by only fructose beverage intake, we performed GO and KEGG pathway enrichment analysis for the above 1978 exclusively up-regulated and 224 exclusively down-regulated DEGs, respectively. The top 20 GO terms of the upregulated and downregulated DEGs are shown in Figures 5A, B. The most significantly upregulated DEGs were related to the GO terms “Cellular metabolic process” and “Intracellular anatomical structure”. The downregulated DEGs were involved in the different GO terms, such as “Oxidative phosphorylation” and ATP synthesis associated signaling pathway.

Subsequently, KEGG pathway analysis demonstrated that the upregulated DEGs were significantly enriched in thyroid hormone signaling pathway, lysosome, endocytosis, autophagy, and protein processing in endoplasmic reticulum (ER) (Figure 5C). Consistent with the GO analysis, KEGG pathway analysis also showed that the most significantly downregulated DEGs were significantly enriched in oxidative phosphorylation (Figure 5D).

### 3.4 PPI network construction and hub gene identification

**Figure 6:**
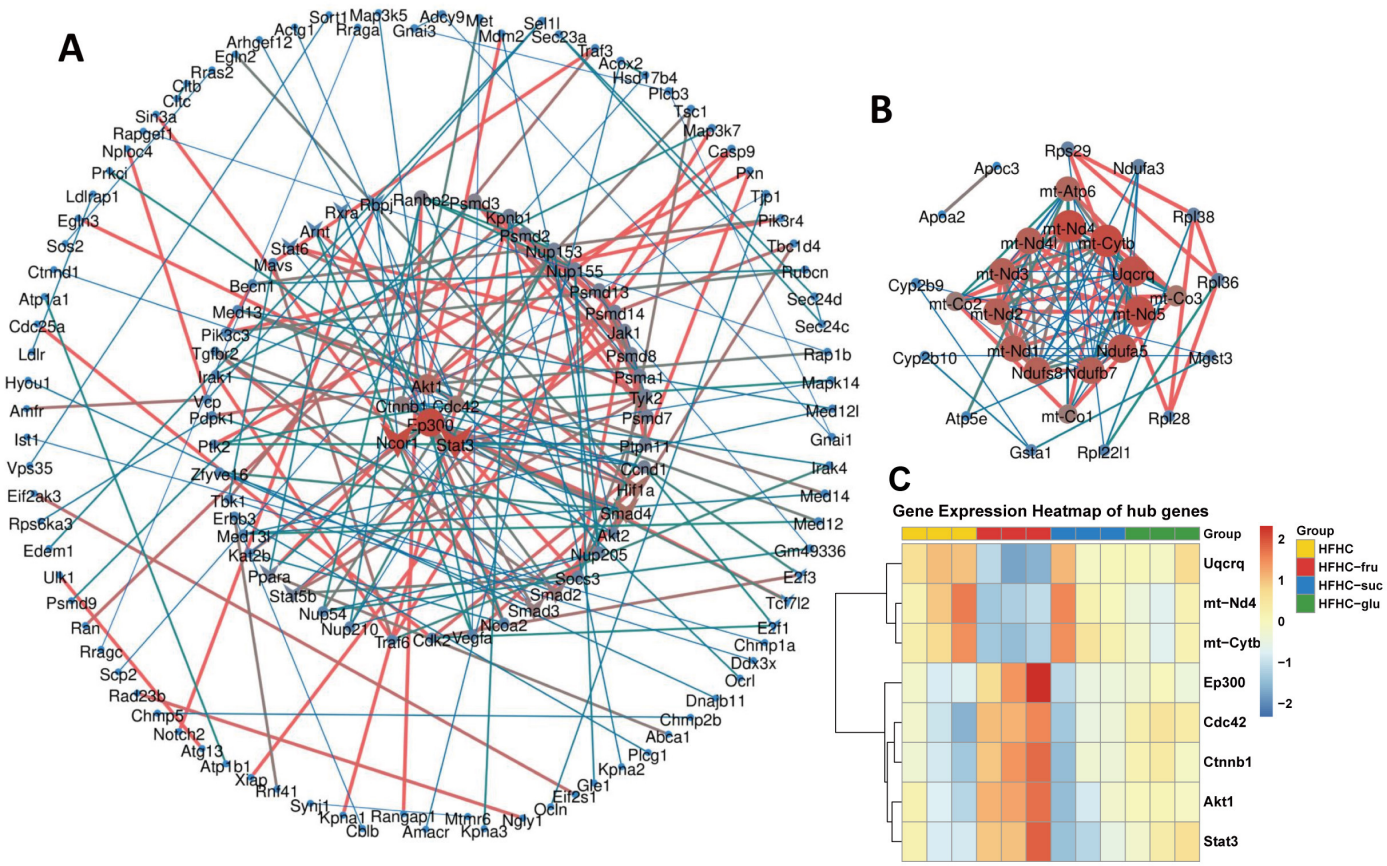
The protein-protein interaction (PPI) network for fructose-specific DEGs. **(A)** The PPI network of 322 exclusively up-regulated DEGs associated with the top 20 KEGG pathways in Figure 5C. **(B)** The PPI network of 47 exclusively down-regulated DEGs associated with the top 20 KEGG pathways in Figure 5D. **(C)** Heatmap showing differential expression of hub genes.

To identify the key genes specifically contributed to fructose metabolism, the 322 upregulated and 47 downregulated DEGs associated with the above top 20 KEGG pathways were imported into the STRING database to obtain the PPI network, respectively. For the upregulated DEGs, the PPI network of upregulated DEGs consisted of 322 gene nodes interacting via 6,395 edges. The top 200 edges (combined score > 969) were shown in Figure 6A. Among these genes, Serine/threonine-protein kinase 1 (Akt1) showed the highest node degree, which was 169. The others included cell division cycle 42 (Cdc42, degree = 131), catenin Beta-1 (Ctnnb1, degree = 113), signal transducer and activator of transcription 3 (Stat3, degree = 100) and E1A Binding Protein P300 (Ep300, degree = 99). The PPI network of the downregulated DEGs included 47 nodes and 222 edges, which top 100 edges (combined score>900) were shown in Figure 6B. We observed that all top hub genes were associated with oxidative phosphorylation signaling pathway, including mitochondrially encoded NADH dehydrogenase 4 (mt-Nd4), mitochondrially encoded cytochrome b (mt-Cytb) and ubiquinol-cytochrome C reductase complex III subunit VII (Uqcrq). Transcriptome sequencing revealed that the expression levels of the five hub genes (Akt1, Stat3, Ctnnb1, Cdc42, and Ep300) were upregulated, while mt-Nd4, mt-Cytb, and Uqcrq were downregulated (Figure 6C, Supplementary Table 1).

**Figure 7:**
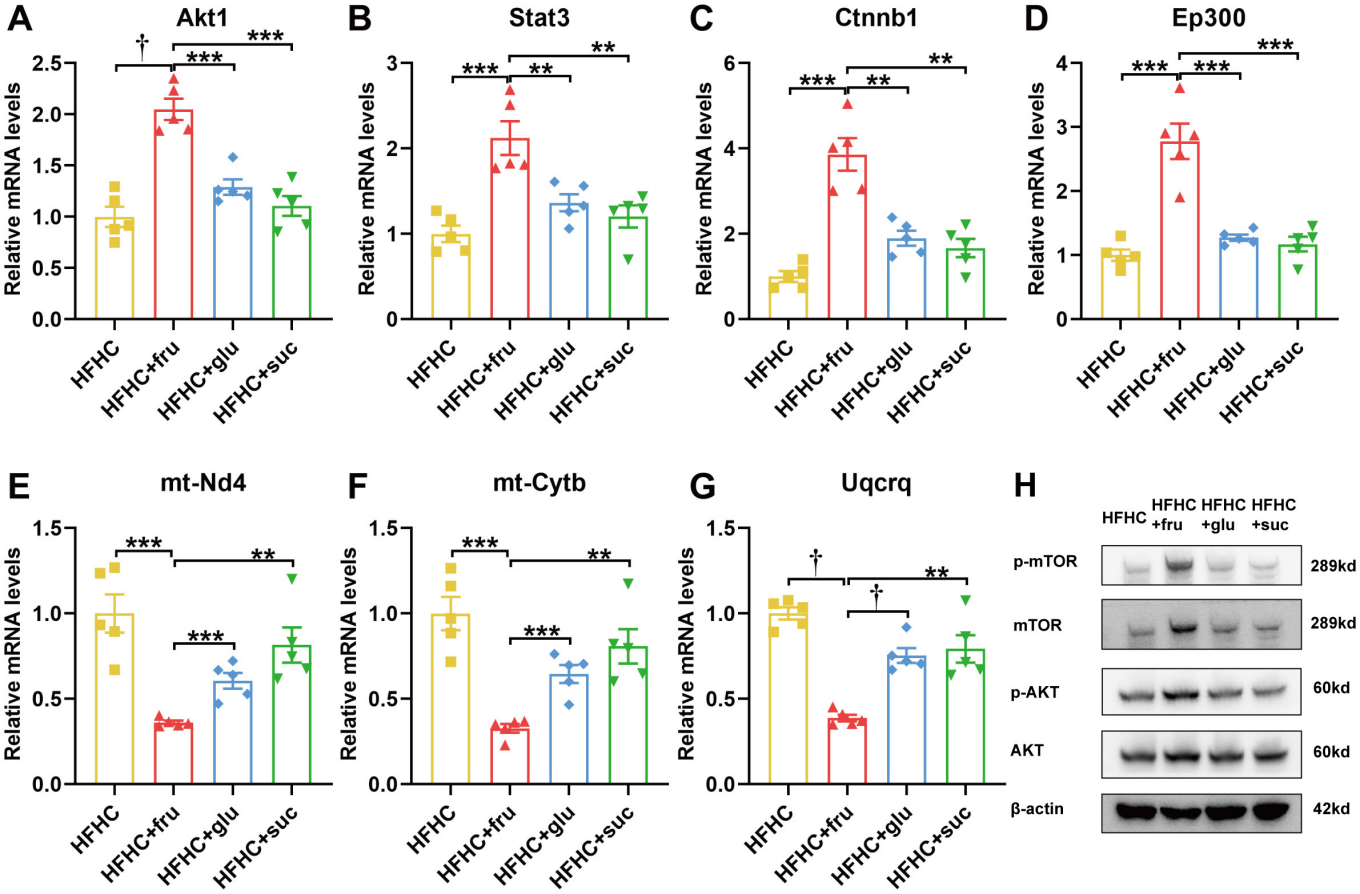
Fructose, but not glucose and sucrose-sweetened beverages disturb the hub gene expression. **(A–G)** The mRNA levels of these indicated genes were measured by RT-qPCR. *n* = 5. **(H)** Protein expression levels of hub genes. Data are mean ± SD. *p < 0.05; **p < 0.01; ***p < 0.001; ^†^*p* < 0.0001; NS, no significance.

Furthermore, we performed real-time qPCR to investigate the hub gene expression in HFHC mice with or without different types of sugar water supplement. Compared to HFHC diet alone, fructose-, but not glucose- and sucrose-SB consumption increased the mRNA levels of Akt1, Stat3, Ctnnb1 and Ep300 (Figures 7A–D). Whereas the expression of mt-Nd4, mt-Cytb and Uqcrq were decreased in mice with consuming beverages sweetened with fructose (Figures 7E–G). Additionally, western blotting confirmed that protein levels of AKT and its downstream effector mTOR were elevated in the livers of mice consuming fructose-sweetened beverages, demonstrating that fructose, relative to sucrose and glucose, more robustly activates hepatic AKT signaling (Figure 7H). Taken together, these results suggested that the above hub genes were critical and exclusive for fructose metabolism.

### 3.5 Single-cell transcriptomic analysis and validation of hub genes

**Figure 8:**
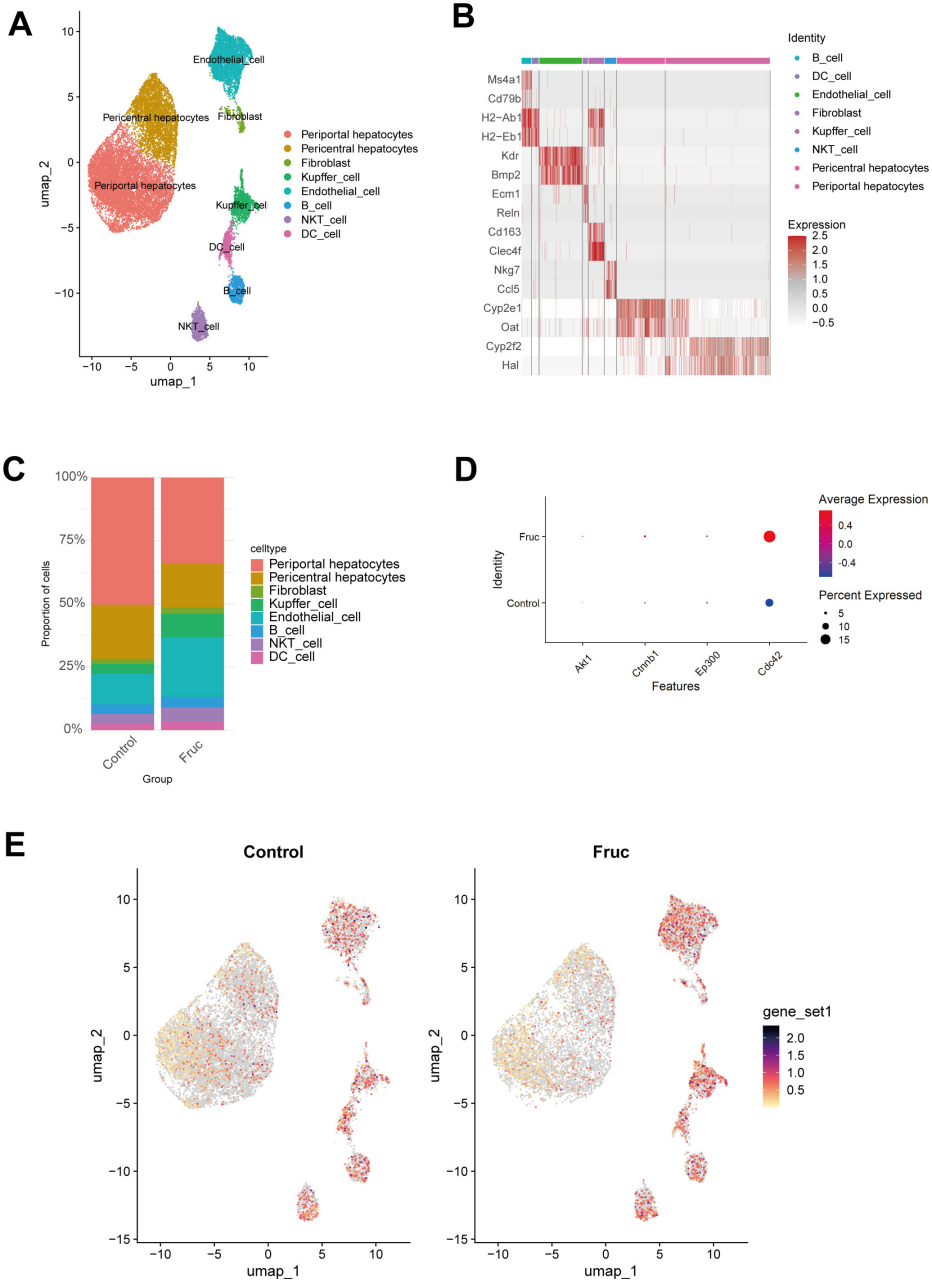
Increased fructose intake elevates hub gene expression validated at the single-cell level. **(A)** UMAP plot showing clustering of single cells into distinct populations. **(B)** Heatmap displaying the top two marker genes for cell-type identification. **(C)** Bar plot of cell-type composition in Control and Fructose groups. **(D)** Dot plot showing differential expression of hub genes between Control and Fructose groups. **(E)** Feature Plot visualization of hub gene expression differences between Control and Fructose groups.

To further investigate and validate the role of hub genes in the effect of high-fructose intake on MASLD, we analyzed the single-cell RNA-seq dataset GSE208750, comparing the high-fructose diet group with the Chow group. Using established marker genes, we identified eight major cell types: B cells, dendritic cells (DCs), endothelial cells, fibroblasts, Kupffer cells, NKT cells, pericentral hepatocytes, and periportal hepatocytes (Figures 8A, B). Notably, the proportions of Kupffer cells and endothelial cells were significantly increased in the high-fructose group (Figure 8C). Consistent with our bulk transcriptome findings, the hub genes Akt1, Ctnnb1, Ep300, and Cdc42 were upregulated in the high-fructose group, with particularly prominent expression changes observed in Kupffer cells and endothelial cells (Figures 8D, E). These findings underscore the critical role of these hub genes in fructose-induced MASLD, highlighting their cell type-specific upregulation and reinforcing their importance in mediating the metabolic effects of dietary fructose.

## 4 Discussion

This study compared the different effects of consuming fructose-, glucose-, and sucrose-SBs in the baseline of HFHC diet fed mice on body weight, liver pathology, liver damage, glucose tolerance, insulin resistance and transcriptome. We demonstrated that intaking fructose-, but not glucose- and sucrose-SB aggravated liver lipid accumulation, liver damage and insulin resistance. Furthermore, we first identified several key upregulated genes (Akt1, Stat3, Ctnnb1 and Ep300) and down regulated genes (mt-Nd4, mt-Cytb and Uqcrq) exclusively in fructose-SB supplement.

It is well-known that consumption of sugar-SBs is a risk factor for the development of T2DM and MASLD (12). However, the different roles of fructose-, glucose-, and sucrose-SBs in weight gain and insulin resistance are controversial. Moreover, less is known about the effects of different sugar-SBs on subjects with MASLD. Herein, HFHC diet was used to induce MASLD mouse model. At the same time, each cage of mouse was given the same volume of water or sugar-SBs. The results revealed that consuming fructose-SBs induced the highest weight gain and liver lipid deposition. Compared to glucose- and sucrose-SB, fructose-SBs enhanced the expression of Acly and Scd1 (Figures 1E, F), two critical enzymes in fatty acid synthesis and metabolism. These data are consistent with the previous point that fructose is distinct from glucose in promoting hepatic de novo lipogenesis (9, 13, 14). Furthermore, consistent with the results of a double-blinded parallel arm study in overweight humans, insulin sensitivity is significantly decreased in subjects consuming fructose but not in those consuming glucose (8). In addition, fructose-SB enhanced HFHC-induced serum ALT and AST, whereas decreased HDL level, which had long been considered as “good cholesterol” for cardio-vascular health. These findings demonstrated that consumption of fructose-SB might associated with a higher risk of MASLD and its complications compared to glucose-containing beverages. The key incremental contribution of our study lies in its direct, side-by-side comparison of fructose, glucose, and sucrose on a MASLD-sensitizing HFHC background, a comparative design notably scarce in recent literature. Our results confirm that fructose exerts a uniquely potent pro-MASLD effect even when compared to its parent disaccharide (sucrose). Strikingly, sucrose-SB did not significantly worsen the MASLD phenotype compared to the HFHC control, suggesting that the form (free vs. bound) and metabolic context critically influence fructose's hepatotoxicity, a nuance highlighted by our head-to-head comparison.

Up to date, publications have reported several signaling pathways involved in high fructose-induced MASLD, such as sterol regulatory element-binding transcription factor 1 (Srebp) and carbohydrate-responsive element-binding protein (ChREBP) mediated de novo lipogenesis (15), oxidative stress (16), mTOR-autophagy-ER stress pathway (17). Our results from KEGG pathways enriched analysis showed that the fructose exclusively up-regulated genes mainly participated in autophagy and protein processing in ER. Interestingly, we found thyroid hormone signaling pathway was associated with fructose function in MASLD progression. Previous study revealed that thyroid hormone signaling could promoted hepatic lipogenesis through the ChREBP (18). Besides, fructose consumption was reported to induce thyroid-related gene expression in brown adipose tissue (19). Thus, it is reasonable to hypothesize that thyroid hormone signaling plays an important role in fructose trigged hepatic lipogenesis. The top 1 enriched signaling pathway of downregulated DEGs is oxidative phosphorylation (OXPHOS), which is considered to be a promising therapeutic target for MASLD (20). Solis-Herruzo et al. (21) had reported that the activity of OXPHOS was significantly decreased in patients with NASH. OXPHOS was also defective in mice fed with high-fat diet and ob/ob mice with MASLD (22, 23). In this study, we found OXPHOS, including the associated hub genes (mt-Nd4, mt-Cytb and Uqcrq), were specifically defective in mice with fructose-SB consumption. A major advance of our study is the deep transcriptomic analysis that moves beyond established mechanisms like DNL. We identified 2,195 fructose-specific DEGs. While the concept of fructose promoting mitochondrial dysfunction has been discussed, our study provides crucial clarity by identifying the specific downregulation of key OXPHOS components (mt-Nd4, mt-Cytb, Uqcrq). To our knowledge, this is the first study to pinpoint these specific molecular lesions within Complex I and III as a unique consequence of fructose-SB, contrasting it with other sugars.

Our findings highlight that Protein kinase B subtype 1 (Akt1) plays a unique and complex dual role in fructose-induced hepatic metabolism, with mechanisms distinct from those observed in glucose metabolism. Fructose, through its unique metabolic pathway that bypasses the key rate-limiting step mediated by phosphofructokinase-1 (PFK-1), provides an unrestricted substrate flux for hepatic *de novo* lipogenesis (DNL) (24). Under acute exposure, fructose can activate Akt1 in an insulin-independent manner and suppress gluconeogenesis through phosphorylation of FoxO1 (25). However, in the context of chronic high-fructose intake, its potent lipogenic capacity induces lipotoxicity, which impairs the insulin-Akt1 signaling pathway and leads to insulin resistance, thereby shifting the primary regulation of DNL to alternative pathways such as ChREBP (26). Consequently, the dynamic response of Akt1 to fructose and the divergence in its downstream metabolic routing fundamentally stem from the unique biochemical metabolic route of fructose, underscoring its central and specific role in fructose-related metabolic diseases. Nevertheless, direct evidence demonstrating the interaction between fructose and Akt1 remains lacking. Thus, further investigation using cell- or tissue-specific knockout models is warranted to elucidate the underlying mechanisms.

The Ctnnb1 gene, which encodes β-catenin, plays important roles in liver homeostasis, NAFLD, and tumorigenesis (27). The genetic polymorphisms in Ctnnb1 gene were reported to affect tumor development, therapeutic responses, and survival in patients with hepatocellular carcinoma (HCC) (28, 29). Furthermore, various studies demonstrated the complexity of Wnt/β-catenin signaling pathway in MASLD progression. The canonical Wnt/β-catenin pathway was reported to function as an anti-inflammatory and anti-lipid accretion signaling (30). However, β-catenin could promote the transcription of lipid synthesizing genes through binding with T-cell factor/lymphoid enhancement factor (TCF) in the non-canonical pathway. Thus, the complexity of the role of β-catenin in MASLD depends on its interactions with different downstream Wnt signal regulators (31). The current study found that Ctnnb1 was significantly exclusively upregulated in MASLD mice with fructose-SB supplement. One of Wnt/β-catenin pathway prominent functions is its role in insulin resistance (32). These findings indicate that Ctnnb1 may play a central role in the pathogenesis of Fructose-induced exacerbation of MASLD.

Our other key hub-genes, Ep300 is a histone acetyltransferase, which functions as transcriptional co-activator protein for CBP (33). It was found that the Ep300-C/EBPα signaling pathway was activated in NAFLD patients. Knockdown of p300 in aged mice inhibited hepatic steatosis (34). Recently, Ep300 histone acetyl-transferase was reported to be a therapeutic target for treatment of liver fibrosis (35). Herein, we firstly found that consumption of fructose-SB induced the expression of Ep300. Further study is required to illuminate the mechanism of action of Ep300 in the progression of fructose-triggered MASLD. The final and perhaps most significant contribution is the identification of a core set of previously unlinked hub genes (Akt1, Stat3, Ctnnb1, Ep300) through our PPI network analysis. Our unbiased approach is the first to identify Stat3, Ctnnb1 (β-catenin), and Ep300 as key hub genes specifically in the fructose-driven aggravation of MASLD, suggesting fructose engages a broad pro-inflammatory and pro-fibrotic signaling network not activated by other sugars. Furthermore, by integrating single-cell data, we demonstrated that the expression of these hub genes was particularly elevated in non-parenchymal cells, a novel insight providing cellular resolution to fructose's deleterious effects.

Importantly, the upregulated and downregulated genes not only work alone, but also interact with each other. For example, activation of Akt1 increased the expression of IL-6, thereby promoting the phosphorylation and activation of Stat3 in non-small cell lung cancer (NSCLC) cells (36). Moreover, STAT3 positively regulated the transcriptional level of β-catenin in colorectal cancer (CRC) cells. In addition, Akt1, Stat3 and Ep300 were reported to play important roles in oxidative phosphorylation (37–39). Our KEGG enrichment analysis and PPI network showed that Ctnnb1 interact with Ep300 and Akt1, are commonly enriched in thyroid hormone signaling pathway. The above analysis indicates that the combined effects of the hub-genes on MASLD are more significant than a single gene.

Based on these findings, it is necessary to acknowledge certain limitations of our study that also point to valuable future research directions with significant clinical implications. Firstly, although we have identified key hub genes (Akt1, Stat3, Ctnnb1, and Ep300) through transcriptomic and network analyses, subsequent functional validation through gene knockout or overexpression experiments was not performed. Such mechanistic studies are crucial to definitively establish the causal roles of these genes in fructose-aggravated MASLD and to assess their potential as novel therapeutic targets for the precise intervention of fructose-driven metabolic diseases. Secondly, given the well-documented sexual dimorphism in MASLD, where prevalence, severity, and progression rates are significantly higher in males, our study exclusively utilized male mice. Consequently, whether the promoting effect of fructose on MASLD progression exhibits sexual dimorphism and the underlying mechanisms remain unexplored. This gap is clinically pertinent, as understanding potential sex-specific responses to dietary fructose is essential for developing tailored nutritional guidelines and gender-informed therapeutic strategies for MASLD patients.

## 5 Conclusion

In conclusion, this study demonstrates that fructose-sweetened beverage consumption uniquely exacerbates HFHC diet-induced MASLD, aggravating hepatic lipid deposition, liver injury, and insulin resistance in a manner distinct from glucose or sucrose. Through transcriptomic analysis, we identified key signaling pathways, including the specific suppression of oxidative phosphorylation (OXPHOS), and pinpointed a novel regulatory network of hub genes that orchestrate the pathological response to fructose. These findings provide direct comparative evidence and unveil novel molecular targets, significantly advancing our understanding of sugar-specific effects on liver health and establishing a valuable foundation for further investigating the distinct pathogenesis of fructose-triggered MASLD.

## Data Availability

The datasets presented in this study can be found in online repositories. The names of the repository/repositories and accession number(s) can be found in the article/Supplementary material.

## Supplementary material

Supplementary Figure S1 Western blot original gel images.

Supplementary Table 1 Transcriptomic sequencing data and results of differential expression analysis for hub genes.
