# Supplementary figures and images for: Identification of the role of sugar-sweetened beverages in the progression of a murine metabolic dysfunction-associated steatotic liver disease model
Source: Front Nutr. 2025 Dec 3;12:1710267. doi: 10.3389/fnut.2025.1710267 (PMC12708248; doi:10.3389/fnut.2025.1710267)

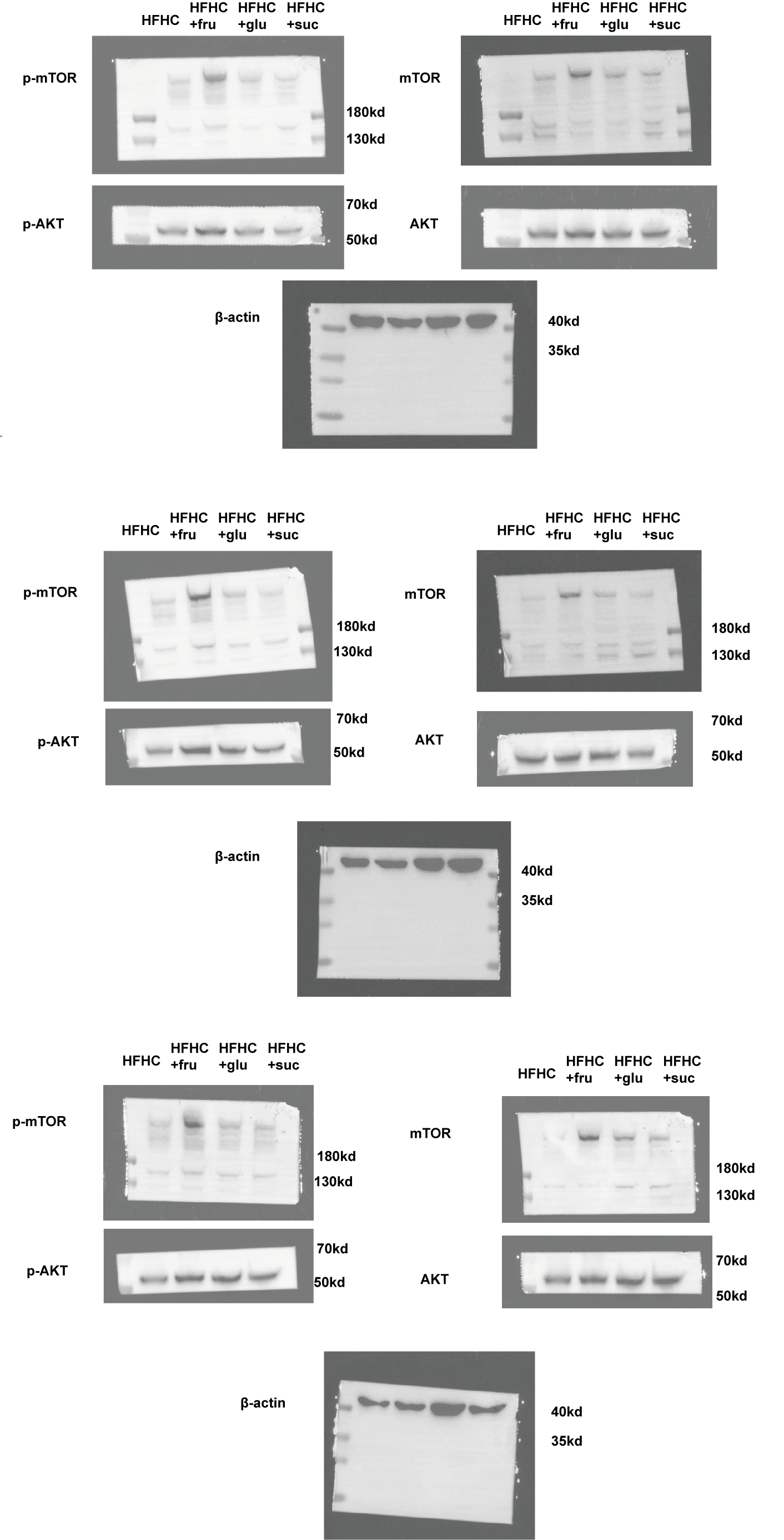

Supplement: Supplementary Figure S1 — Western blot original gel images. [file Image_1.TIF]
